# Compensators: An alternative IMRT delivery technique

**DOI:** 10.1120/jacmp.v5i3.1965

**Published:** 2004-10-21

**Authors:** Sha X. Chang, Timothy J. Cullip, Katharin M. Deschesne, Elizabeth P. Miller, Julian G. Rosenman

**Affiliations:** ^1^ University of North Carolina Medical School Department of Radiation Oncology Chapel Hill North Carolina 27514 U.S.A.; ^2^ Forsyth Memorial Hospital Department of Radiation Oncology Winston‐Salem North Carolina 27103 U.S.A.

**Keywords:** compensator, IMRT, dose optimization, QA, treatment time

## Abstract

Seven years of experience in compensator intensity‐modulated radiotherapy (IMRT) clinical implementation are presented. An inverse planning dose optimization algorithm was used to generate intensity modulation maps, which were delivered via either the compensator or segmental multileaf collimator (MLC) IMRT techniques. The in‐house developed compensator‐IMRT technique is presented with the focus on several design issues. The dosimetry of the delivery techniques was analyzed for several clinical cases. The treatment time for both delivery techniques on Siemens accelerators was retrospectively analyzed based on the electronic treatment record in LANTIS for 95 patients. We found that the compensator technique consistently took noticeably less time for treatment of equal numbers of fields compared to the segmental technique. The typical time needed to fabricate a compensator was 13 min, 3 min of which was manual processing. More than 80% of the approximately 700 compensators evaluated had a maximum deviation of less than 5% from the calculation in intensity profile. Seventy‐two percent of the patient treatment dosimetry measurements for 340 patients have an error of no more than 5%. The pros and cons of different IMRT compensator materials are also discussed. Our experience shows that the compensator‐IMRT technique offers robustness, excellent intensity modulation resolution, high treatment delivery efficiency, simple fabrication and quality assurance (QA) procedures, and the flexibility to be used in any teletherapy unit.

PACS numbers: 87.53Mr, 87.53Tf

## I. INTRODUCTION

The most common techniques today for delivering IMRT treatments on linear accelerators use multileaf collimators (MLCs).^(1)^ The obvious benefit of the MLC‐based intensity‐modulated radiotherapy (IMRT) techniques is treatment delivery automation. The MLC leaves move automatically during the treatment of each field to form the intensity modulation and between fields to define treatment ports, saving radiation therapists trips into the treatment room. It is widely believed that the automation of MLC‐based IMRT technique simplifies the treatment delivery relative to nonautomated intensity‐modulated treatment techniques. Years of clinical application, however, have shown that the increased technical and mechanical complexity of MLC‐IMRT techniques weakens the benefit gained by automation. MLC‐based IMRT techniques have shown several drawbacks in clinical application.^(^ ^2^ ^–^ ^13^ ^)^ For instance, the total monitor units (MUs) required for a segmental MLC‐IMRT treatment are often much higher than that of the corresponding nonintensity modulated treatment. As a result, the treatment delivery time is often considerably extended, and concerns about radiation contamination of the prolonged beam‐on time are also raised.^(9)^ The often highly irregularly shaped MLC segment fields pose challenges to dose and MU calculation.^(14)^ The dynamics of intensity map production by the MLC‐IMRT techniques might also interfere with the dynamics of patient organ motion when it is considered for treatment planning.^(15)^

An alternative way to deliver the intensity‐modulated treatment is by using a physical compensator. Compensators have been used in radiotherapy for decades to produce simple forms of intensity modulation. As the sophistication level of radiotherapy treatment planning and delivery techniques improved over the years, so did compensator techniques and their application. In the last decade compensator techniques have been used for delivering IMRT treatments designed by dose optimization algorithms.^(^ ^2^ ^,^ ^16^ ^–^ ^26^ ^)^ Customized compensators are shaped to attenuate the open‐field photon fluence such that the transmitted fluence map is as designed by the dose optimization algorithm. The obvious advantage of this IMRT delivery method is simplicity. The static nature of the compensator intensity modulation simplifies the treatment delivery, dose computation, and thus the quality assurance (QA) procedure. Another advantage of the compensator‐IMRT technique is that it can create continuously varying intensity modulation, whereas the intensity modulation created by an MLC‐based technique is discrete at least in one direction. One obvious drawback of most of the compensator‐IMRT techniques is the lack of automation. Radiation therapists need to go into the treatment room and exchange customized compensators between treatment fields. Recently, methods that automate the compensator exchange between treatment fields have been developed to improve the treatment delivery efficiency.^(^ ^23^ ^,^ ^26^ ^)^ Another common concern for compensator‐IMRT is the fabrication and assembly time, which has been reported to be extensive for some compensator techniques.^(^ ^2^ ^,^ ^21^ ^,^ ^28^ ^,^ ^43^ ^)^ In reviewing the aforementioned literature, we found that there is a large variation in the reported cost and in intensity map resolution of compensator‐IMRT techniques.

It is important to separate the issue of intensity modulation design, which is primarily performed by the dose optimization algorithm, from the issue of how a given intensity modulation pattern is delivered. A desirable IMRT delivery technique should be able to faithfully reproduce the intensity maps given, independent of how they are designed and thus independent of the dose optimization algorithm used. The focus of this paper is an intensity modulation delivery technique.

We began to develop the compensator‐IMRT technique in 1993 before any accelerator MLC systems were commercially available. We have routinely used the compensator technique to deliver intensity‐modulated treatments for over 700 patients since 1996. Although we have published some of our compensator‐IMRT work in the past,^(^ ^16^ ^,^ ^29^ ^,^ ^30^ ^)^ the details of our technique have never been published. Recently, there has been a renewed interest from the radiation therapy community in this IMRT delivery technique. A number of commercial treatment‐planning system vendors have provided users the option of delivering IMRT treatments via compensator by interfacing their treatment‐planning system to automated milling machines for intensity map output. Nonetheless, we find compensator‐IMRT remains a less‐understood concept for many today. There are widespread concerns and even misconceptions about the compensator‐IMRT delivery techniques on issues ranging from efficiency to quality to cost. We hope this paper will provide a better understanding of compensator‐IMRT techniques and their value as alternative techniques for IMRT treatment delivery.

## II. METHODS

### A. Dose optimization algorithm and intensity map generation and delivery

**Figure 1:**
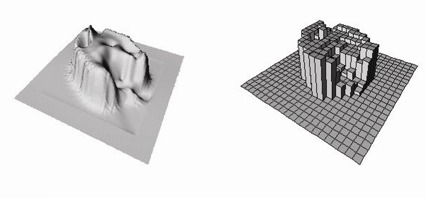
An ideal intensity map (left) produced by index‐dose gradient optimization for a head and neck treatment with a multistructure objective. IMRT‐compensator is generated from the ideal intensity map. For segmental MLC‐IMRT treatment a discrete intensity map (right) is converted from the ideal map for MLC segmentation. The discrete “skyscraper” map displayed has 10 intensity levels.

An in‐house developed inverse planning algorithm based on index‐dose gradient minimization^(30)^ is used for treatment plan optimization. The optimization objective is multistructural and dose volume histogram (DVH) based and has been implemented in our in‐house treatment‐planning system, PLanUNC (PLUNC). The details of the dose optimization algorithm have been previously published.^(30)^ A typical clinical fluence map generated by the dose optimization algorithm is shown in Fig. 1(a). The fluence maps are continuous and smooth, since the limitations of the actual treatment delivery technique are not considered in the optimization, except for the maximum range of the intensity modulation. The smooth fluence map treatment plan represents the ideal treatment, which we use as the “gold standard” to evaluate the quality of an actual deliverable IMRT treatment. The actual treatment is delivered using either the compensator or the segmental MLC delivery technique. For the MLC treatment delivery, PLUNC truncates the smooth fluence map into a skyscraper‐like discrete map with a given number of intensity levels and spatial resolution. Figure 1 (right) shows a 10 intensity level discrete fluence map, the highest level used in our clinical application, generated from the smooth map (Fig. 1 (left)).

In our clinical application five to eight intensity levels are commonly used. The IMFAST ^(31)^ MLC segmentation optimization software (Siemens Medical Systems, Inc., Concord, CA) converts the discrete intensity maps to MLC segments. The resulting MLC segments are exported back to PLUNC for final dose computation and plan evaluation. Once the plan is approved, it is downloaded to the LANTIS record & verify system (Siemens Medical Systems). The treatments are delivered on Siemens accelerators with MLC via the Primeview/SimTec system (Siemens Medical Systems). When the compensator technique is chosen in PLUNC, the smooth fluence map is directly converted to a compensator file in a format specified by the automated milling machine (Par Scientific, Model ACD‐3, Odense, Denmark). Once the dose optimization is complete, the planner decides which IMRT delivery technique to use for patient treatment after evaluating both the dosimetric quality and the treatment efficiency of each technique. The dosimetric quality is compared to that of the ideal treatment and the optimization objective; the total number of segment fields governs the treatment efficiency. Several segmental IMRT techniques with a different number of IM levels were normally assessed.

### B. Compensator design and material selection

#### B.1 Compensator thickness design

The compensator thickness, $t^{\text{comp}}(x,y)$ traversed by the pencil beam is determined from the attenuation equations below with the consideration of beam divergence and beam hardening.

(1) $$I^{IMRT}(x,y)=I^{open}(x,y)e^{-\mu \cdot t^{comp}(x,y)}$$

*where*

(2) $$\mu =\mu_{0}+c_{1}\cdot t^{comp}(x,y)+c_{2}\cdot r+c_{3}\cdot S$$

where $I^{\text{IMRT}}$ and $I^{\text{open}}$ represent the desired modulated fluence and the otherwise open field fluence, respectively. The (*x, y*) coordinate system is defined at the bottom plane of the compensator when placed in the wedge slot in the accelerator head (*x* and *y* are consistent with the orientations of *x* and *y* collimators in the accelerator). The linear attenuation coefficient of the pencil beam in the compensator material is denoted by μ, and the path length of the beam in the material is $t^{\text{comp}}$. Parameters *S* and *r* in Eq. (2) represent the treatment field size (with an equivalent square field of S×S) and the off‐axis distance, respectively. The second term in the equation represents “beam hardening” of the pencil beam going through the compensator material. The third term is the beam energy variation at off‐axis distance *r.* PLUNC considers the pencil beam energy change at different off‐axis distance caused by the flatness filter. The last term in Eq. (2) is the effective attenuation coefficient change for the pencil beam due to the scattered photons generated in the compensator. These equations describe the attenuation of the photon pencil beam intensity, not dose, through the compensator material.

**Figure 2:**
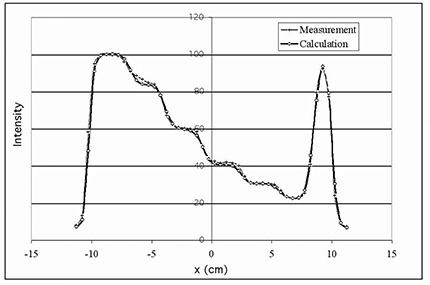
Profile comparison for a step‐like tungsten powder compensator for compensator‐IMRT commissioning. The pencil beam attenuation parameters described in Eqs. (1) and (2) are manually and iteratively adjusted to achieve the best fit between the measured and calculated intensity profiles.

The constants in the equations, $\mu_{\tilde{0}}$ and $c_{n})(n=\;1,\;2,3)$, are manually and iteratively determined by fitting the calculated to the measured test profiles. Several step‐like test compensators were used for fitting of the attenuation coefficients. A 25‐cm square field, which is larger than all test compensators in each dimension, was used for the profile comparison. In each field there is a range of pencil beam intensity attenuation magnitudes—no attenuation for the pencil beams not going through the compensator and varying attenuation for beams going through the compensator. Figure 2 shows the measurement and the final calculation after the fitting for a step‐like test compensator of the tungsten powder material. The actual fitting was performed at different depths since the value of the attenuation coefficient is known to vary with field size and measurement depth. The optimal numerical values for parameters $\mu_{0}^{\sim }({\text{cm}}^{-1})$ and $c_{1}\;({\text{cm}}^{-2})$ were previously published ^(16)^ for the tin granule compensator material. Table 1 lists the attenuation parameters we derived from test measurements for both of the compensator materials at two photon beam energies. The parameter $c_{3}$ in the last term in Eq. (2) is negligible for the medium density tin granule compensator. For the high‐density tungsten powder compensator there are more scattered photons from the compensator, which causes a decrease of approximately 10% in the value of $\underset{˙}{\mu }$ from a field size of 5 cm×5 cm to 20 cm×20 cm.

**Table 1:** Photon beam pencil beam attenuation coefficients (see Eqs. (1) and (2)) of two compensator materials

Energy	Compensator material	$\mu_{0}({\text{cm}}^{-1})$	$c_{1}({\text{cm}}^{-1})$	$c_{2}({\text{cm}}^{-1})$
6 MV	tin granules tungsten powder	0.217 0.430	0.005 0.019	0.001 0.004
15 MV	tin granules tungsten powder	0.175 0.365	0.003 0.009	0.005 0.002

Once $t^{\text{comp}}(x,y)$ is determined using Eqs. (1) and (2), the compensator thickness file can be calculated. For a milling machine with vertical movements only the compensator thickness $t^{\prime }(x^{\prime },y^{\prime })$ is designed so that the pencil beam going though (*x, y*) traverses a distance of $t^{\text{comp}}(x,y)$ in the compensator material. The angle between the central axis and the pencil beam is $\underset{˙\;˙\;˙}{\mu }$ and *D* is the distance from the photon source to the (*x, y*) plane. The compensator file parameters are calculated using the following equations:

(3) $$t^{\prime }(x^{\prime },y^{\prime })=t^{comp}(x,y)\cdot \cos\theta$$

(4) $$x^{\prime }=x\cdot (1-\frac{t^{comp}(x,y)}{D}\cos\theta )$$

(5) $$y^{\prime }=y\cdot (1-\frac{t^{comp}(x,y)}{D}\cos\theta )$$

This thickness determination method for nondiverging milling machine drill bit is an approximation that can produce errors where adjacent pencil beam intensities differ drastically. The effect of this error combined with that of a finite drill bit size on intensity map generation is reflected in the intensity map QA (IMRT QA) for each treatment field. In our years of practice of using both compensator and MLC‐based IMRT delivery techniques, we have found that the IM map QA quality of compensator‐IMRT treatments is not inferior to that of the segmental MLC‐IMRT treatments.

### B.2 Compensator spatial resolution

PLUNC samples 64×64 infinitesimal pencil beams in intensity map calculation independent of the field size. The intensities of other pencil beams are derived via linear interpolation. The sampling frequency, however, can be increased at the cost of computation time. Because PLUNC generates smoothly varying intensity maps without high spatial frequency variations,^(30)^ we have not noticed significant issues with sampling resolution. The largest symmetric field dimension achievable by our current compensator‐IMRT design is 25 cm cross‐plane and 30 cm in‐plane.

**Figure 3:**
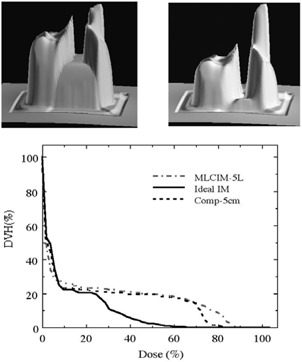
Intensity map from the dose optimization with 5 cm compensator (tin granule compensator) thickness limit (top left); intensity map of the same optimization without the compensator depth limitation (top right); and the cord DVH comparison for the treatment plans with (Comp‐5cm) and without the depth limitation (Ideal IM) (bottom). The cord DVH result of a segmental MLC‐IMRT plan (MLCIM‐5L) is also displayed.

**Figure 4:**
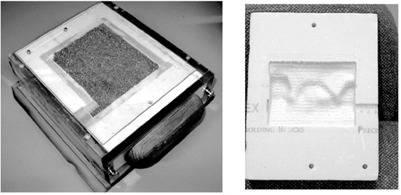
Compensator box with a tin granule‐filled compensator enclosed (left) and a Styrofoam compensator mold (right). The three reference holes on the mold and the matching set on the box are used for easy verification of the compensator orientation in the box. The compensator is designed to be inserted in the wedge slot of an accelerator.

**Figure 5:**
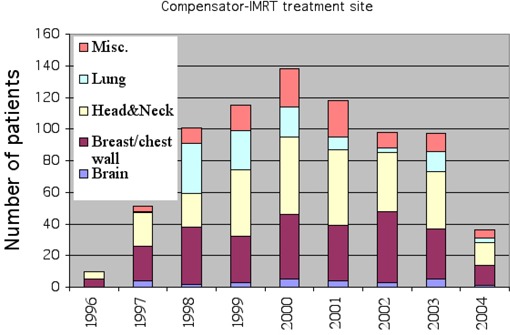
Summary of the number of patients treated per year using the compensator‐IMRT technique broken down by treatment site from November 1996 to May 2004.

**Figure 6:**
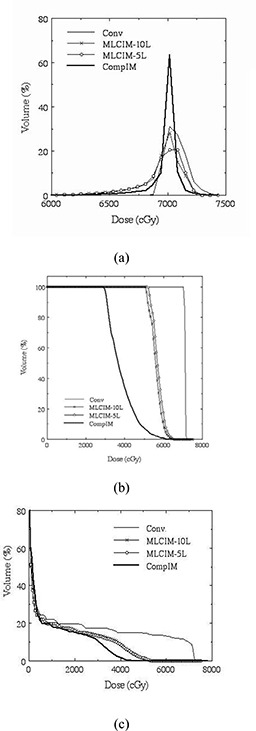
DVH comparison of the compensator‐based (solid line) and the segmental MLC‐based IMRT treatments as well as the corresponding conventional treatment (dashed line) for a six‐field nasopharynx tumor treatment. Intensity levels of 5 (open circle line) and 10 (solid square line) were used in creating the MLC‐based IMRT treatment. (a) PTV (differential DVH), (b) chiasm, and (c) cord.

**Figure 7:**
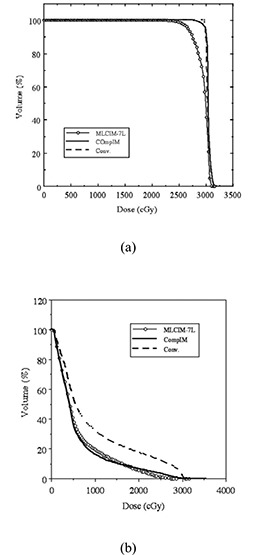
DVH of PTV (a) and rectum (b) for a five‐field prostate treatment. The compensator‐based (solid line) and the segmental MLC‐based (line with circles) IMRT treatment are shown as well as the corresponding conventional treatment (dashed line). Seven intensity levels were used in converting the ideal intensity map to the discrete map for the MLC‐based treatment.

**Figure 8:**
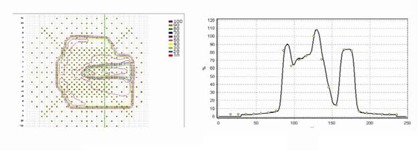
Typical examples of compensator‐IMRT QA result by MapCheck 2D detector system (left) and Profiler 1D detector system (right). The solid line is calculated and the open circle is measured data. The two exemplary measurements are not related.

The computer‐controlled compensator milling setup takes the drill radius (3 mm) into consideration. Effectively, a 6‐mm wide “sliding window” averaging is applied to the calculated compensator topography along the direction of the milling path. In the worst‐case scenario, where the field dimension perpendicular to the milling path is 25 cm, the resolution perpendicular to the milling path is 3.9 mm, less than the drill bit diameter of 6 mm. A detailed theoretical analysis on the influence of the drill size and other factors on the accuracy of compensator‐generated intensity modulation was carried out by Meyer et al.^(2)^ The finite drill size is not considered in the dose optimization. However, the error in compensator‐generated intensity map due to milling limitation is reflected and evaluated in the IMRT QA procedure. We have compared the intensity maps of compensators made using drill bits of 3 mm, 2.5 mm, and 1.75 mm radius and found that the differences between the compensator‐produced intensity maps are small for the cases tested (see Figs. 2 to 8).

### B.3 Compensator margin

The main purpose of using a margin is to accommodate our clinical need to make small field edge modifications during the course of treatment. We have extended the compensator to cover a margin outside the treatment portal to anticipate any positive field size change/error that is less than 1.5 cm. The compensator pattern outside the treatment portal is an extension of the compensator pattern at the nearest field edge. When the needed field size change is positive and no more than 1 cm, the original compensator can often be used. When the intended change is a reduction of the field dimension, the same compensator can generally be used. Once the compensator is made, it is treated as a customized wedge in PLUNC, and the dosimetry of a changed treatment plan can be easily recalculated. Regardless of the sign of the field size change, the new dosimetry is recomputed and reviewed. The planner then decides if the original compensator is adequate for the changed treatment.

### B.4 Compensator IM range limit

The density of compensator material and its maximum thickness limit the intensity modulation range achievable by a compensator. In our design the maximum thickness of the compensator is 5 cm. The maximum intensity modulation range achieved by the tin granule compensators is approximately 100% to 38% and 45% of the open field for 6 MV and 15 MV, respectively. This modulation range does not always meet our clinical needs. This can be demonstrated in a clinical case whose optimization objective includes PTV (planning target volume) dose uniformity and sparing of the spinal cord nearby. Figure 3 shows two different intensity maps of the same field generated using the same optimization but with different IM range limits. (Figure 3top left) shows the intensity map generated with the 5 cm compensator depth limitation and (Fig. 3top right) without the depth limitation. The “filled” valley (where cord lies beneath) in the intensity map in (Fig. 3top left) indicates that the intended intensity modulation, shown in (Fig. 3top right), is much larger than what is actually achievable by this compensator. The resulting dosimetry in Fig. 3(bottom) is consistent with the difference seen in the intensity map, showing suboptimal results of the 5 cm compensator depth in the cord DVH comparison. The cord would be overdosed if the compensator‐IMRT method was used. In cases like this, we often select the MLC‐based technique, which has the largest intensity modulation range, from 100% to the leakage through the collimators. However, the limited spatial and intensity resolution of the segmental MLC‐IMRT technique with 5 IM levels (MLCIM‐5L) can sometimes counteract the advantage of its large intensity modulation range as shown in Fig. 3(bottom). MLC‐IMRT plans using more IM levels (10 and 7) did not improve the cord DVH because the limited spatial resolution (1 cm×1 cm) is responsible for the cord dosimetry deterioration.

Recently, we have investigated the use of a coarse tungsten powder as a compensator material. Tungsten powder has an effective density of more than $10{g/\text{cm}}^{3}$ compared to $4.6\;{g/\text{cm}}^{3}$ for tin granules. With the same compensator design tungsten offers a much larger intensity modulation range: 100% to 18% and 20% for 6 MV and 15 MV, respectively. Based on our experience, we believe this new intensity modulation range is adequate for most of our dose optimization needs. Note the compensator is used for intensity modulation generation only; the treatment portal is defined by either MLC or blocks.

### B.5 Compensator material selection

There are a number of materials that have been used to form compensators, including lead,^(^ ^32^ ^–^ ^35^ ^)^ Cerrobend^(36)^ brass,^(37)^ aluminum,^(37)^ steel,^(38)^ tin^(16)^ tin‐wax mixture,^(39)^ gypsum,^(40)^ Lucite,^(41)^ and tungsten‐epoxy mixture.^(27)^ The physical form of the material ranges from powders to granules, small cubes to solids and mixtures. The following are what we consider the criteria for the ideal compensator material and physical form to generate smooth intensity modulation:

• large range of intensity modulation magnitude

• intensity modulation of high spatial resolution

• not hazardous for handling in the fabrication process

• easy to form to and retain the shape needed

• low material cost

• friendly to the environment

The first criterion calls for compensator materials of high density and/or large thickness compensator design. The second criterion prefers powder, granule, or other nondiscrete physical forms of compensator materials. Table 2 lists several material choices and their pro and cons for compensator application.

**Table 2:** Pros and cons of selected materials for the IMRT compensator application

Material	Pro	Con
Cerrobend (with and without mold)	• readily available • inexpensive • recyclable • high density	• need a milling machine
brass/steel/lead (cube or sheet)	• no milling required • recyclable • inexpensive	• poor IM resolution due to discreteness • can be labor‐intensive for assembly. • can be hazardous (lead)
Lucite (solid)	• easy to machine • nonhazardous	• low density thus low IM magnitude • need a milling machine • not recyclable thus can be expensive
brass/steel (solid)	• readily available • can produce smooth IM • nonhazardous	• not recyclable thus can be expensive • need a milling machine
tin granule‐wax (mixture in mold)	• recyclable • can produce smooth IM • nonhazardous	• low density thus low IM magnitude • need a milling machine • difficult to keep consistent packing density
tin/steel (granule in mold)	• high IM resolution • consistent packing • nonhazardous • recyclable	• medium density ‐medium IM magnitude • need a milling machine
tungsten (powder in mold)	• high IM resolution • consistent packing • high density • recyclable	• slightly hazardous to handle in coarse powder form (less than Cerrobend and lead) • need a milling machine

In the past, Cerrobend has not been considered an excellent compensator material despite its large density. Cerrobend shrinks from its liquid form in the compensator mold when it solidifies, potentially causing significant deviation from the intended compensator shape and density uniformity. We recently found that there are Cerrobend filling techniques that produce smooth and accurate compensators with consistent density, as demonstrated by Par Scientific. Thus solidified Cerrobend in the compensator mold becomes one of the top choices of compensator material. The other good choice for compensator in mold is coarse tungsten power. Recyclable tungsten powder has an effective density close to that of Cerrobend (approximately $10\;\text{grams}/{\text{cm}}^{-3}$), and it can be easily shaped to the intended form with uniform density using the technique described below.

### C. Compensator assembly

The compensator mold is milled out of a Styrofoam block. Figure 4 shows the sturdy and easy‐to‐handle compensator box that is easily inserted into the wedge slot of a teletherapy unit. The compensator box ensures the integrity of the compensator throughout the treatment and guarantees compensator alignment to the radiation beam. The tin granules and the box are reused; only the Styrofoam mold is discarded. The same compensator box can be used on different accelerators of the same wedge slot design, with and without an MLC device, to deliver the IMRT treatment.

The compensator box is designed to prevent human errors by offering no options in application and to provide easy and clear identification of assembly error. For each compensator, a QA sheet is generated in PLUNC to be used as the reference for the compensator mold fabrication QA measurement. A medical physics technician performs the compensator fabrication, assembly, and geometric QA. The technician verifies and records the shape and orientation of the compensator mold pattern, the maximum depth of the compensator, and the distance from each compensator edge to the edge of the Styrofoam mold using the compensator QA sheet as the reference.

Milling a set of reference holes is a useful function offered by the software of the milling machine. The location, diameter, and depth of the reference holes are fixed and independent of the compensator shape and are used to check errors in milling machine operation. After the compensator mold passes the above inspection, it is enclosed by a 2 mm‐thick Lexan sheet. The enclosed mold is filled and packed with tin granules through a small hole drilled on the side. A household electric muscle massager was used in filling to ensure compacted packing and a consistent tin granule density for all compensators. The visual sign of the packing status is quite clear: The tin granules “dance” in the mold under vibration until the mold is tightly packed. The tin granule‐filled Styrofoam compensator mold is then labeled with the patient and field names before being inserted into the acrylic compensator box.

Prior to initial clinical implementation, the compensator packing density consistency was carefully studied. A 2% maximum variation in compensator weight was observed for nearly 50 different packings by different operators at different times following the same packing instruction. The 2% weight variation in the tin granule compensator would cause a pencil beam intensity variation of 0.4% per centimeter of compensator thickness, assuming the weight variation occurs uniformly.

### D. Compensator‐IMRT dosimetry QA

Clinical physicists perform the remaining tasks in the compensator‐IMRT QA procedure. This consists of the verification of the intensity map under a test phantom condition and of patient treatment dose measurement. The verification of the intensity map was performed using a 1D diode array system Profiler and, recently, a 2D array diode system MapCheck (both systems are from Sun Nuclear Corporation, Melbourne, Florida). A 25 cm×25 cm field size (which is larger than most of the compensator covered fields, at least in 1D) is used for the Profiler measurement. When the MapCheck 2D diode array system is used, the actual treatment fields are used for QA measurement. PLUNC calculates the 1D (for Profiler) and 2D (for MapCheck) dose distribution in the QA phantom from the treatment IM field and exports the dose map to Profiler/MapCheck system. At the time of data collection the measured and calculated intensity maps are displayed and compared in the MapCheck or Profiler software. Both the relative and absolute doses are compared. We found in our Profiler QA data that the differences between measured and calculated compensator factors are less than 2% at dmax and less than 3% at 15 cm depth. Clinical examples of the IMRT QA and the statistics of the discrepancy between the measurement and calculation in our clinical application are presented in the Results section.

For each compensator‐IMRT treatment patient, treatment dosimetry was measured before the first 10 Gy of dose was delivered. MOSFET detectors (Thomson & Nielsen Electronics Ltd., Ottawa, ON, Canada) are placed on the entrance or exit portal of the selected IM fields. Bolus of 1 cm thickness is used for the measurement. Whenever possible, a MOSFET dosimeter is placed in the field where the intensity fluence variation is relatively slow. The calculated dose MOSFET received at the measurement location was verified in PLUNC by the physicist who conducted the MOSFET measurement. The uncertainty of the calculated skin dose is estimated to be 10%. The rapid dose change and the lack of dose calculation accuracy in this region by the pencil beam dose calculation algorithm, plus the fact that the bolus perturbs the measurement, make it difficult to obtain accurate dose verification. The statistics of patient measurements are presented in the Results section.

### E. Treatment technique comparison methods

The compensator‐based and the segmental MLC‐based IMRT techniques are compared in terms of the treatment efficiency and dosimetry. The treatment delivery time data are based on the daily patient treatment record in LANTIS treatment record & verify system; the dosimetry of the treatment technique is computed and compared in PLUNC. Chang et al.^(16)^ and Potter et al.^(42)^ have shown that the dose optimization quality of the ideal treatment with smooth intensity maps is generally higher compared with the corresponding MLC‐IMRT treatments by IMFAST segmentation. In IMFAST,^(31)^ the platform algorithm with fluence correction was used in the segmentation. In their systematic study of the dosimetric quality and treatment efficiency of IMFAST segmentation algorithm for head and neck treatments Potter et al.^(42)^ have shown that the selection of the segmentation methods available in IMFAST has limited influence on the dosimetry quality and the treatment delivery efficiency.

For the treatment time analysis the finishing time stamp for the compensator‐IMRT treatment is at the “beam‐on” time of the last field, and of the last segment for the MLC‐IMRT treatment. The treatment completion time is not recorded by LANTIS. The last compensator field generally requires more MUs, thus more time, than the last MLC segment field. This average systematic offset in the treatment delivery time analysis is estimated to be no more than 30 s for a 180 cGy treatment at 200 MUs/min. In the analysis of the compensator‐IMRT treatment time we have adjusted the treatment time recorded in LANTIS by adding the treatment time of the last compensator field, which is calculated based on the number of MUs and the accelerator output rate. No such adjustment is made for the MLC‐IMRT treatment time. The LANTIS treatment delivery time record automatically includes the time therapists spent between treatments of different fields to exchange compensators.

## III. RESULTS

### A. Treatment case summary

Over 700 patients have been treated using the compensator‐IMRT delivery technique since November 1996. Figure 5 shows the breakdown of the number of cases per treatment site. The three major treatment sites are head and neck, breast/chestwall, and lung from November 1996 to April 2004. The decrease in the number of compensator‐IMRT cases starting in 2001 is associated with the beginning of clinical implementation of segmental MLC‐IMRT in our clinic.

### A.1 Optimization quality comparison

The effect of intensity modulation resolution of IMRT delivery techniques can be demonstrated in the following two clinical examples. The first example is a six‐field nasopharynx tumor treatment whose optimization objective includes PTV dose uniformity and sparing of multiple nearby critical structures. Figures (6a), (b), (c) show a DVH comparison of a six‐field nasopharynx treatment for the PTV and two critical structures between the smooth intensity map (CompIM) plan and the discrete map plans. Two intensity resolutions (or IM levels) were used in converting the original smooth map to the corresponding discrete maps for MLC segment optimization, and resulting plans are named MLCIM‐5L and MLCIM‐10L. The result of the corresponding manually planned conventional treatment, using the same beams without intensity modulation, is also displayed in Fig. 6 for reference.

The figures clearly illustrate that the resolution of the delivered intensity maps can have a significant impact on the quality of the treatment optimization, especially when the optimization objective includes sparing of small critical structures close to the treatment volume. For the segmental MLC‐IMRT technique, the increase in intensity resolution alone (IM level) did not result in noticeable improvements in dosimetry for critical structures chiasm and cord (see Figs. 6(b), (c)) in this case. This indicates that the spatial resolution, which is governed by the size of the MLC leaf, is likely responsible for the dosimetric deterioration from the ideal IMRT treatment.

Figure 7 displays a similar comparison for a five‐field prostate treatment. The optimization objectives are PTV dose uniformity and DVH‐specified rectal sparing. In this case, the differences between the continuous IM technique and the discrete IM technique are reflected in the PTV. The rectal sparing is similar, but PTV dose uniformity of the discrete MLC‐IM technique was worse.

The above two clinical examples show that intensity modulation resolution of an IMRT delivery technique can have a significant impact on the quality of the optimized treatment the patient receives. The finer resolution compensator‐IMRT technique can produce intensity modulation that is closer to the ideal intensity modulation compared to segmental MLC‐based IMRT techniques.

### A.2 Intensity modulation QA

The software‐driven diode array system Profiler and recently MapCheck are used for compensator IMRT QA. Profiler measures one beam profile at a time, typically, about 5 min per compensator, including the time for equipment setup and comparison with calculation (in MapCheck software). The calculated intensity profiles are exported to the MapCheck software computer ahead of time, and they are used to verify the measured intensity profile during the data collection. This real‐time QA feature is very desirable in our busy clinic. The static nature of the compensator intensity modulation allows us to use only a few MUs (10 MUs are sufficient although 50 MUs are used) for the measurement. MLC‐IMRT techniques, on the other hand, dictate that the actual treatment MUs be used for the QA measurement. We analyzed the maximum deviation of the measured profile relative to the calculated profile for more than 1600 clinical compensator profile scans.

The maximum discrepancy between the calculated and the measured profiles within the treatment field (with 1.5‐cm margin) is recorded in the QA procedure. We found that 83% of the compensators had a maximum discrepancy between measured and calculated intensity profile of 0% to 5%, 16% of the compensators had a discrepancy of 5.1% to 10%, and 1% had a discrepancy of 10.1% to 15% compared to the calculation. The profile comparison error in regions outside the treatment field can be larger due to the limitations of the milling machine generated compensator as analyzed by Meyer et al.^(2)^ The larger point dose discrepancies between calculation and measurement in the IMRT QA often (but not always) occur at IM map regions of high gradient. In comparison, we found that statistically the agreement between calculation and measurement for compensator‐IMRT is noticeably better than segmental MLC‐IMRT. We found that the MLC leaf positioning accuracy is the main source of error in segmental MLC‐IMRT QA. Figure 8 shows examples of the intensity map validation for an IMRT compensator using MapCheck and Profiler.

### A.3 Patient dosimetry QA

MOSFET dosimeters are placed on user‐specified entrance points for selected IM fields. Bolus of 5 mm to 10 mm thickness is used, depending on availability. The measured dose is then compared with the calculation on PLUNC. We analyzed the results of 340 patients and found that 71% of them had a measurement‐calculation discrepancy of no more than 5%, 26% of them had a discrepancy between 5.1% and 10%, and 3% of the patients had a discrepancy of more than 10%. The exact effect of bolus on the patient surface cannot be simulated in PLUNC, and there are rapid dose changes at superficial depths; thus, the dose calculation uncertainty at skin is estimated to be 10%.

### A.4 Treatment delivery time

We retrospectively analyzed patient treatment timing information in LANTIS treatment record & verify system. The treatment delivery time is defined as the time elapsed from the beginning of the first field/segment irradiation to the end of the last field/segment irradiation. We found the typical variation in daily treatment time, excluding port film days, was one minute for both the compensator and segmental MLC‐based IMRT deliveries. The LANTIS record & verify system showed that compensator‐IMRT delivery time for a typical five‐field prostate treatment was less than 5 min. For a similar treatment (five‐field and 180 cGy per fraction) the LANTIS record showed that segmental MLC‐IMRT technique is more than 10 min. Our compensator‐IMRT deliveries are significantly shorter than the compensator‐IMRT delivery time reported by Levegrün et al,^(43)^ where 12 min were required for five‐field compensator‐IMRT treatment, a time that is even longer than the time for today's segmental MLC technique. The treatment delivery time improvement brought about by IM‐MAXX, a segmental MLC‐IMRT delivery enhancing option on Siemens accelerators, is also presented below.

**Figure 9:**
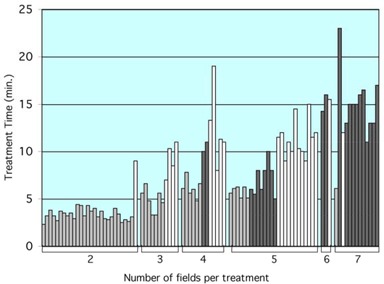
Patient treatment delivery time for the compensator‐IMRT treatments (gray) and the segmental MLC‐IMRT treatments. MLC‐IMRT treatments with the IM‐MAXX option (black) and the MLC‐IMRT treatments without IM‐MAXX option (white) are also shown. The data are retrospectively analyzed from the LANTIS record & verify system recorded patient treatment delivery information.

Figure 9 displays LANTIS‐recorded treatment delivery time for IMRT treatments of 95 patients. The treatment time is averaged over the entire treatment course excluding the portfilm days. The delivery techniques used were MLC‐IMRT treatments with and without IM‐MAXX and compensator‐IMRT treatment. The number of treatment fields per patient ranges from 2 to 7. For MLC‐IMRT treatments, the number of intensity levels used was generally between 5 and 7, although the latter was more common. There are some variations in prescription dose between 180 cGy and 200 cGy, accelerator MU rate between 200 MU/min and 500 MU/min, and treatment site. We estimated that these variations would cause a variation in treatment time of less than one minute. These variations in the data should have no influence on the trend of the treatment delivery time in Fig. 9.

Clearly, there are other factors contributing to the variation seen in the patient treatment delivery time record shown in Fig. 9. For instance, some of the treatment times for the four‐field treatments are longer than those for five‐field treatments for all types of IMRT treatments. We speculate that this could be related to the stability of patient treatment setup and patient condition. When therapists notice large patient movement during treatment, more time can be spent on patient interaction and repositioning. The figure shows that the compensator‐IMRT technique requires the least treatment time in comparison with the MLC techniques for almost all patients. The lack of automation of the compensator technique is well compensated by its delivery efficiency.

**Figure 10:**
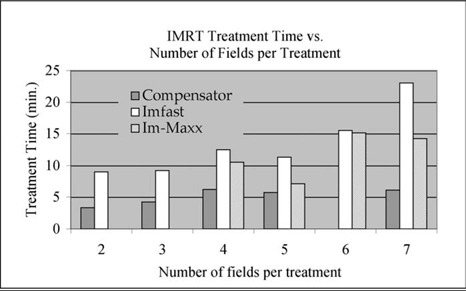
The average treatment delivery time for IMRT treatments with different numbers of fields. The average is taken over the treatments of the same IMRT type and total number of treatment fields.

Figure 10 shows the average treatment delivery time for IMRT of a different number of treatment fields. The treatment time here is averaged over the treatments of the same number of treatment fields shown in Fig. 10. Our data indicate that when the field number increased from 4 to 7, the treatment delivery time increased 2.5 times for segmental MLC‐IMRT treatment without IM‐MAXX option; 1.3 times for the compensator‐IMRT treatments; for MLC‐IMRT treatment with IM‐MAXX option, the treatment time increased 1.5 times. On average, the reduction in segmental MLC‐IMRT treatment time by IM‐MAXX is 23.5%. Figure 10 shows that the compensator technique is clearly the fastest technique in our routine clinical use.

### A.5 Compensator fabrication time

The typical time for compensator fabrication including the time for fabrication‐related QA procedures is about 13 min, 10 min of which is milling machine time. The time the medical physics technician actually spends on compensator assembly and QA is normally only 3 min per compensator (the time for a physicist to perform IMRT QA was discussed previously). Considering the significant savings of treatment delivery time every day as shown in Fig. 10, we consider 13 min of compensator fabrication to be time well spent. The time required for compensator disassembly and recycling after treatment completion is less than 2 min per compensator.

## IV. DISCUSSION

We realize that some compensator techniques require significantly longer time for fabrication and assembly than our technique. For instance, Levegrün et al.^(43)^ reported that it normally took 4 h to fabricate five compensators. Their compensators were made of lead sheet layers; we speculate that the manual assembly process for this type of compensator is time‐consuming. There are several other factors that contribute to the efficiency of compensator production. The functionality of the milling machine can certainly make a major difference in the milling time. Styrofoam is also much easier and quicker to mill than a solid metal. Another contributing factor is the design of the compensator box, which requires minimal effort in manual alignment and assembly. The in‐house resource for computer programs and interfaces played an instrumental role in the development and evolution of the streamlined program.

A general limitation for compensator‐IMRT techniques is the intensity modulation range, which is directly related to the compensator material density and thickness. When medium density material is used, such as granules of tin or steel, the maximum range of intensity modulation can be a significant limiting factor in delivering an optimized treatment. Granules do have some valuable advantages (see Table 2) compared with the solid form. However, they only have 52% of the linear attenuation coefficient of the solid form, if the granules are identical spheres. One way to increase the packing density is to mix granules of different sizes and shapes.

We did not consider the mechanical constraints of the MLC or the resolution constraints of the compensator technique in the dose optimization. We speculate that consideration of these constraints in dose optimization may improve the dosimetric quality of the treatments, especially for segmental MLC‐IMRT treatment, as discussed by Siebers et al.^(44)^ The MLC‐IMRT results reported here apply only to segmental MLC‐IMRT delivery by Siemens accelerators. We speculate that the treatment efficiency for the fast‐moving “sliding window” type MLC‐IMRT delivery by Varian accelerators would be closer to that of the compensator‐IMRT. This manuscript is intended as a report on our experience in developing and implementing a compensator‐IMRT technique in our clinic.

In addition to its robustness and simplicity, another important advantage of the compensator‐based technique is its ability to produce fine intensity modulation resolution. This in turn generates high spatial resolution treatment dosimetry. Many published compensator‐IMRT techniques unnecessarily limit themselves to the same resolution as the MLC‐based techniques. We speculate that one of the reasons is that treatment‐planning systems are designed for discrete MLC IMRT techniques We have shown in this and our previous work that intensity modulation resolution can play a very important role in preserving the quality of dose optimization. We hope to see more compensator‐IMRT techniques make the most of this inherent advantage.

As we make further advances in treatment planning and delivery technology, consideration of patient intra‐fraction motions, such as organ motion, during treatment optimization becomes feasible. The shorter treatment time and the static nature of the compensator‐IMRT technique can be further beneficial when organ motion is considered. Zygmanski et al.^(7)^ and Chui^(45)^ have reported that compensator‐IMRT techniques can bypass some of the difficulties encountered by MLC‐based techniques in dose optimization that considers organ motion.

## V. CONCLUSION

We have shown that our compensator‐IMRT technique has several benefits for delivering continuous intensity modulation. Our seven years of clinical application experience demonstrate that the robust compensator‐IMRT delivery system is efficient in terms of fabrication, assembly, QA, and treatment delivery. We have shown that the finer resolution compensator‐IMRT technique can also produce dosimetry that is closer to the ideal IMRT treatment (without any delivery limitation) compared with the segmental MLC IMRT technique.

## ACKNOWLEDGMENT

We wish to thank Larry Potter for his helpful assistance in the graphical data presentation. We are grateful to Dr. Joel Tepper for his constructive comments in the preparation of this manuscript. Finally, we want to thank our colleagues all over the world who have shown strong interest in and support of this work.
